# Supplementary material for: Human Endocrine-Disrupting Effects of Phthalate Esters through Adverse Outcome Pathways: A Comprehensive Mechanism Analysis
Source: Int J Mol Sci. 2023 Aug 31;24(17):13548. doi: 10.3390/ijms241713548 (PMC10488033; doi:10.3390/ijms241713548)
Supplement: Supplementary file 1 [file ijms-24-13548-s001.zip › Supplementary Material.pdf]

Supplement Materials

Human Endocrine-Disrupting Effects of Phthalate Esters through Adverse Outcome Pathways: A Comprehensive Mechanism Analysis

Yunxiang Li †, Hao Yang †, Wei He and Yu Li *

College of Environmental Science and Engineering, North China Electric Power University, Beijing 102206, China; 120222232055@ncepu.edu.cn (Y.L.); 120212232080@ncepu.edu.cn (H.Y.); 120222132018@ncepu.edu.cn (W.H.)

* Correspondence: 50201549@ncepu.edu.cn

† These authors contributed equally to this work.

```
import numpy as np
import pandas as pd
import matplotlib.pyplot as plt
pd.set_option('display.max_rows',None)
pd.set_option('display.max_columns',None)
data=pd.read_excel('C:\\Users\\WIN11\\Desktop\\study\\cjs.xlsx')
X=data.iloc[:,2:]
Y=data.iloc[:,1]
from sklearn.model_selection import train_test_split
x_train,x_test,y_train,y_test=train_test_split(X,Y,test_size = 0.3,random_state=0)
from xgboost import XGBRegressor
import xgboost as xgb
from sklearn.metrics import mean_squared_error
from sklearn.metrics import r2_score
```

```
C:\Users\WIN11\Desktop\1\lib\site-packages\xgboost\compat.py:36: FutureWarning: pandas.Int64Index is deprecated and will be removed from pan
das in a future version. Use pandas.Index with the appropriate dtype instead.
  from pandas import MultiIndex, Int64Index
```

```
for i in range(1,100):
    xgb_model=xgb.XGBRegressor(
        random_state=0,
        n_estimators=30,
        alpha=17,
        eta=1.45,
        subsample=0.66,
        max_depth=10
    )
    xgb_model.fit(x_train,y_train)
    y_pred = xgb_model.predict(x_test)

    score = r2_score(y_test,y_pred)
# 模型得分

    print("i=" +str(i)+"得分",score)
```

```
    xgb_model=xgb.XGBRegressor(
        random_state=0,
        n_estimators=30,
        alpha=17,
        eta=1.45,
        subsample=0.66,
        max_depth=10
    )
    xgb_model.fit(x_train,y_train)
    y_pred = xgb_model.predict(x_test)

    score = r2_score(y_test,y_pred)
R2 = r2_score(y_test,y_pred)
R2
```

```
C:\Users\WIN11\Desktop\1\lib\site-packages\xgboost\data.py:250: FutureWarning: pandas.Int64Index is deprecated and will be removed from pand
as in a future version. Use pandas.Index with the appropriate dtype instead.
  elif isinstance(data.columns, (pd.Int64Index, pd.RangeIndex)):
```

```
0.8443402626244294
```

Figure S1. Estrogen single effect XGboost model code

```python
import pandas as pd
import numpy as np
from scipy.stats import pearsonr
```

```python
dataSet = pd.read_excel("C://Users//WIN11//Desktop//study//lyxsj.xlsx")
pearson_result=dataSet.corr()
pearson_result.to_excel("C://Users//WIN11//Desktop//study//pearson.xlsx")
```

```python
def excuteFilter(x,pearson_result,dataSet,path):
    header=pearson_result.columns
    data = pearson_result.values
    indices = np.triu_indices_from(data)
    [rows, cols] = data.shape
    addlist = []
    removelist = []
    removeindex = []
    for i in range(rows):
        if header[i] not in removelist:
            for j in range(cols):
                if j < i:
                    if data[i,j]>x or data[i,j]<-x:
                        if header[j] in addlist:
                            if header[i] not in removelist:
                                removelist.append(header[i])
                                continue
                        if header[i] not in addlist:
                            addlist.append(header[i])
                        if header[j] not in removelist:
                             removelist.append(header[j])
    retainList = []
    for col in header:
        if col not in removelist:
            retainList.append(col)
            print(col)
    dataSet[retainList].to_excel(path,index = False)
```

```python
excuteFilter(0.75,pearson_result,dataSet,"C://Users//WIN11//Desktop//study//retainDataSet.xlsx")
```

```python
import seaborn as sns
import matplotlib.pyplot as plt
dataframe = pd.read_excel("C://Users//WIN11//Desktop//study//retainDataSet.xlsx")
dcorr=dataframe.corr()
plt.rcParams['font.sans-serif']=['Times New Roman']
plt.rcParams['axes.unicode_minus']=False
plt.rcParams['xtick.labelsize']=20
plt.rcParams['ytick.labelsize']=20
plt.subplots(figsize=(30, 30))
fig=sns.heatmap(dcorr,
            cmap='Purples',
            annot=True,
            annot_kws={'size':20},
            fmt=".2f",
            mask=np.triu(np.ones_like(dcorr,dtype=bool))
           )
fig_path="C://Users//WIN11//Desktop//study//retainHeatmap.jpg";
heatmap = fig.get_figure()
heatmap.savefig(fig_path, dpi = 400)
```

Figure S2. Pearson correlation coefficient model code

```python
import numpy as np
import pandas as pd
import matplotlib.pyplot as plt
pd.set_option('display.max_rows',None)
pd.set_option('display.max_columns',None)
data=pd.read_excel('C:\\Users\\WIN11\\Desktop\\study\\22.xlsx')
X=data.iloc[:,2:]
Y=data.iloc[:,1]
from sklearn.model_selection import train_test_split
x_train,x_test,y_train,y_test=train_test_split(X,Y,test_size = 0.3,random_state=0)
```

```python
from sklearn.linear_model import LinearRegression
lr= LinearRegression(
)
from sklearn.metrics import mean_squared_error
from sklearn.metrics import r2_score
lr.fit(x_train,y_train)
y_pred = lr.predict(x_test)
R2 = r2_score(y_test,y_pred)
R2
```

```
-22444.24575239423
```

Figure S3. Multiple linear regression model code of AOP initial event of PAEs endocrine disrupting effect

```
import numpy as np
import pandas as pd
import matplotlib.pyplot as plt
pd.set_option('display.max_rows',None)
pd.set_option('display.max_columns',None)
data=pd.read_excel('C:\\Users\\WIN11\\Desktop\\study\\33.xlsx')
X=data.iloc[:,2:]
Y=data.iloc[:,1]
from sklearn.model_selection import train_test_split
x_train,x_test,y_train,y_test=train_test_split(X,Y,test_size = 0.3,random_state=0)
```

```
from sklearn.linear_model import LinearRegression
lr= LinearRegression(

)
from sklearn.metrics import mean_squared_error
from sklearn.metrics import r2_score
lr.fit(x_train,y_train)
y_pred = lr.predict(x_test)
R2 = r2_score(y_test,y_pred)
R2
```

```
-1363.8238250551356
```

Figure S4. Multiple linear regression model code of AOP key event of PAEs endocrine disrupting effect

```
import numpy as np
import pandas as pd
import matplotlib.pyplot as plt
pd.set_option('display.max_rows',None)
pd.set_option('display.max_columns',None)
```

```
data=pd.read_excel('22.xlsx')
X=data.iloc[:,2:]
Y=data.iloc[:,1]
from sklearn.model_selection import train_test_split
x_train,x_test,y_train,y_test=train_test_split(X,Y,test_size = 0.3,random_state=0)
from sklearn.ensemble import RandomForestRegressor
from sklearn.metrics import mean_squared_error
from sklearn.metrics import r2_score
```

```
for i in range(1,100):
    RF = RandomForestRegressor(
         n_estimators=2,
        criterion = 'mae',
        random_state=3,
        max_samples=10,
        max_depth=4,
    )
    RF.fit(x_train,y_train)
    y_pred = RF.predict(x_test)

    score =r2_score(y_test,y_pred)
# 模型得分

    print("i=" +str(i)+"得分",score)
```

```
    RF = RandomForestRegressor(
        n_estimators=2,
        criterion = 'mae',
        random_state=3,
        max_samples=10,
        max_depth=4,

    )
    RF.fit(x_train,y_train)
    y_pred = RF.predict(x_test)
R2 = r2_score(y_test,y_pred)
R2
```

```
C:\Users\WIN11\Desktop\1\lib\site-packages\sklearn\ensemble\_forest.py:403: FutureWarning: Criterion 'mae' was deprecated in v1.0 and will b
e removed in version 1.2. Use `criterion='absolute_error'` which is equivalent.
  warn(
```

```
0.8518272706950072
```

Figure S5. random forest regression model
code of AOP initial event of PAEs endocrine disrupting effect

```
import numpy as np
import pandas as pd
import matplotlib.pyplot as plt
pd.set_option('display.max_rows',None)
pd.set_option('display.max_columns',None)
data=pd.read_excel('C:\\Users\\WIN11\\Desktop\\study\\33.xlsx')
X=data.iloc[:,2:]
Y=data.iloc[:,1]
from sklearn.model_selection import train_test_split
x_train,x_test,y_train,y_test=train_test_split(X,Y,test_size = 0.2,random_state=0)
from sklearn.ensemble import RandomForestRegressor
from sklearn.metrics import mean_squared_error
from sklearn.metrics import r2_score
```

```
for i in range(1,100):
    RF = RandomForestRegressor(
         random_state=0,
         n_estimators=46,
        max_depth=20,
        min_samples_split=8,
        max_features=4
    )
    RF.fit(x_train,y_train)
    y_pred = RF.predict(x_test)

    score =r2_score(y_test,y_pred)
# 模型得分

    print("i=" +str(i)+"得分",score)
```

```
    RF = RandomForestRegressor(
         random_state=0,
         n_estimators=46,
        max_depth=20,
        min_samples_split=8,
        max_features=4
    )
    RF.fit(x_train,y_train)
    y_pred = RF.predict(x_test)
R2 = r2_score(y_test,y_pred)
R2
```

```
0.8548904323288249
```

Figure S6. random forest regression model
code of AOP key event of PAEs endocrine disrupting effect

```
import numpy as np
import pandas as pd
import matplotlib.pyplot as plt
pd.set_option('display.max_rows',None)
pd.set_option('display.max_columns',None)
data=pd.read_excel('C:\\Users\\WIN11\\Desktop\\study\\55.xlsx')
X=data.iloc[:,2:]
Y=data.iloc[:,1]
from sklearn.model_selection import train_test_split
x_train,x_test,y_train,y_test=train_test_split(X,Y,test_size = 0.2,random_state=0)
from sklearn.ensemble import RandomForestRegressor
from sklearn.metrics import mean_squared_error
from sklearn.metrics import r2_score
```

```
for i in range(1,100):
    RF = RandomForestRegressor(
        random_state=20,
        n_estimators=5,
        max_depth=20,
        max_features=5,
        min_samples_split=5
    )
    RF.fit(x_train,y_train)
    y_pred = RF.predict(x_test)

    score =r2_score(y_test,y_pred)
# 模型得分

    print("i=" +str(i)+"得分",score)
```

```
 RF = RandomForestRegressor(
      random_state=20,
        n_estimators=5,
        max_depth=20,
        max_features=5,
        min_samples_split=5
    )
RF.fit(x_train,y_train)
y_pred = RF.predict(x_test)
R2 = r2_score(y_test,y_pred)
R2
```

```
0.7505073863664207
```

Figure S7. random forest regression model
code of AOP adverse ending of PAEs endocrine disrupting effect

```
import numpy as np
import pandas as pd
import matplotlib.pyplot as plt
pd.set_option('display.max_rows',None)
pd.set_option('display.max_columns',None)
```

```
data=pd.read_excel('C:\\Users\\WIN11\\Desktop\\study\\22.xlsx')
X=data.iloc[:,2:]
Y=data.iloc[:,1]
from sklearn.model_selection import train_test_split
x_train,x_test,y_train,y_test=train_test_split(X,Y,test_size = 0.3,random_state=0)
from xgboost import XGBRegressor
import xgboost as xgb
from sklearn.metrics import mean_squared_error
from sklearn.metrics import r2_score
```

```
C:\Users\WIN11\Desktop\1\lib\site-packages\xgboost\compat.py:36: FutureWarning: pandas.Int64Index is deprecated and will be removed from pan
das in a future version.  Use pandas.Index with the appropriate dtype instead.
  from pandas import MultiIndex, Int64Index
```

```
xgb_model=xgb.XGBRegressor(
           n_estimators=17,
           max_depth=2,
           random_state=0,
           eta=0.8,
        gamma=3,
        max_delta_step=61,
    )
xgb_model.fit(x_train,y_train)
y_pred = xgb_model.predict(x_test)
R2 = r2_score(y_test,y_pred)
R2
```

```
C:\Users\WIN11\Desktop\1\lib\site-packages\xgboost\data.py:250: FutureWarning: pandas.Int64Index is deprecated and will be removed from pand
as in a future version.  Use pandas.Index with the appropriate dtype instead.
  elif isinstance(data.columns, (pd.Int64Index, pd.RangeIndex)):
```

```
0.8592365821546417
```

Figure S8. XGboost regression model code of AOP initial event of PAEs endocrine disrupting effect

```
import numpy as np
import pandas as pd
import matplotlib.pyplot as plt
pd.set_option('display.max_rows',None)
pd.set_option('display.max_columns',None)
data=pd.read_excel('C:\\Users\\WIN11\\Desktop\\study\\33.xlsx')
X=data.iloc[:,2:]
Y=data.iloc[:,1]
from sklearn.model_selection import train_test_split
x_train,x_test,y_train,y_test=train_test_split(X,Y,test_size = 0.2,random_state=0)
from xgboost import XGBRegressor
import xgboost as xgb
from sklearn.metrics import mean_squared_error
from sklearn.metrics import r2_score
```

```
for i in range(1,100):
    xgb_model=xgb.XGBRegressor(
        random_state=12,
        n_estimators=20,
        max_depth=20,
        eta=0.4,
        subsample=0.8,
        gamma=4,
        alpha=3,
        colsample_bytree=0.9
    )
    xgb_model.fit(x_train,y_train)
    y_pred = xgb_model.predict(x_test)

    score = r2_score(y_test,y_pred)
# 模型得分

    print("i=" +str(i)+"得分",score)
```

```
xgb_model=xgb.XGBRegressor(
  random_state=12,
        n_estimators=20,
        max_depth=20,
        eta=0.4,
        subsample=0.8,
        gamma=4,
        alpha=3,
        colsample_bytree=0.9
)
xgb_model.fit(x_train,y_train)
y_pred=xgb_model.predict(x_test)
R2 = r2_score(y_test,y_pred)
R2
```

```
C:\Users\WIN11\Desktop\1\lib\site-packages\xgboost\data.py:250: FutureWarning: pandas.Int64Index is deprecated and will be removed from pand
as in a future version.  Use pandas.Index with the appropriate dtype instead.
  elif isinstance(data.columns, (pd.Int64Index, pd.RangeIndex)):
```

```
0.8359270436326218
```

Figure S9. XGboost regression model code
of AOP key event of PAEs endocrine disrupting effect

```
import numpy as np
import pandas as pd
import matplotlib.pyplot as plt
pd.set_option('display.max_rows',None)
pd.set_option('display.max_columns',None)
data=pd.read_excel('C:\\Users\\WIN11\\Desktop\\study\\55.xlsx')
X=data.iloc[:,2:]
Y=data.iloc[:,1]
from sklearn.model_selection import train_test_split
x_train,x_test,y_train,y_test=train_test_split(X,Y,test_size = 0.2,random_state=0)
from xgboost import XGBRegressor
import xgboost as xgb
from sklearn.metrics import mean_squared_error
from sklearn.metrics import r2_score
```

```
for i in range(1,100):
    xgb_model=xgb.XGBRegressor(
        min_child_weight=3,
        max_depth=3,
        subsample=0.69,
        eta=0.5
    )
    xgb_model.fit(x_train,y_train)
    y_pred = xgb_model.predict(x_test)

    score = r2_score(y_test,y_pred)
# 模型得分

    print("i=" +str(i)+"得分",score)
```

```
    xgb_model=xgb.XGBRegressor(
        min_child_weight=3,
        max_depth=3,
        subsample=0.69,
        eta=0.5,
    )
    xgb_model.fit(x_train,y_train)
    y_pred = xgb_model.predict(x_test)
R2 = r2_score(y_test,y_pred)
R2
```

```
C:\Users\WIN11\Desktop\1\lib\site-packages\xgboost\data.py:250: FutureWarning: pandas.Int64Index is deprecated and will be removed from pand
as in a future version.  Use pandas.Index with the appropriate dtype instead.
  elif isinstance(data.columns, (pd.Int64Index, pd.RangeIndex)):
```

```
0.9481267975614578
```

Figure S10. XGboost regression model code
of AOP adverse ending of PAEs endocrine disrupting effect
